# Dynamic Changes in Intestinal Gene Expression and Microbiota across Chicken Egg-Laying Stages

**DOI:** 10.3390/ani14111529

**Published:** 2024-05-22

**Authors:** Kai Shi, Xiangping Liu, Ying Duan, Xusheng Jiang, Ni Li, Yuesong Du, Dongfeng Li, Chungang Feng

**Affiliations:** College of Animal Science and Technology, Nanjing Agricultural University, Nanjing 210095, China; 2020205003@stu.njau.edu.cn (K.S.); 2022805098@stu.njau.edu.cn (X.J.); lidongfeng@njau.edu.cn (D.L.)

**Keywords:** microbiota, intestine, microbiome, transcriptome, chicken egg-laying

## Abstract

**Simple Summary:**

Simple Summary: The production of eggs is a crucial aspect of the chicken industry. Recent research has indicated that the gut microbiota undergoes dynamic changes and plays a significant role in egg production. However, the relationship between the gut microbiota and host gene expression remains unclear. To investigate this, we collected samples from the intestine (i.e., duodenum, jejunum, and ileum) and its microbiota at different stages of egg-laying (i.e., pre-, peak-, and late-laying) in chickens. Our findings showed that intestinal flora underwent significant changes during different laying periods that included changed genes (i.e., *APOA1*, *APOB*, *TST*, *CCDC93*, and *TMEM175*) involved in several transport processes, response to DNA damage, and development of intestinal structure. Additionally, our analysis suggested that specific intestinal microbes were significantly correlated with host gene expression, which indicated an interaction between the gut microbiota and the chicken host.

**Abstract:**

Eggs are a vital dietary component for humans, and it is beneficial to increase egg production to support poultry farming. Initially, the egg production rate rises rapidly with young hens until it reaches its peak, and then it declines gradually. By extending the duration of peak egg production, the hens’ performance can be enhanced significantly. Previous studies found dynamic changes in gut microbiota during egg-laying, and several species of microbiota isolated from the chicken gut improved egg-laying performance. However, the interaction between microbes and host gene expression is still unclear. This study provides a more comprehensive understanding of chicken egg-laying by examining dynamic alterations in the microbiota of the entire intestinal tract (i.e., duodenum, jejunum, and ileum) and gene expression. The microbial community in the intestine underwent significant changes during different egg-laying periods (i.e., pre-, peak-, and late-laying periods). Metagenomic functional analysis showed that the relative abundance of biosynthesis of amino acids, secondary metabolites, and cofactors decreased significantly in the duodenum, jejunum, and ileum of aging hens. The relative levels of aldosterone, GnRH, insulin, growth hormone, and other hormone-related pathways increased dramatically in the intestinal microbiota during egg-laying, but only in the microbiota located in the duodenum and ileum. Transcriptome analysis suggested that genes associated with various transport processes were upregulated consistently in the small intestine during egg-laying; genes involved in the development of intestinal structure were down-regulated; and genes involved in response to DNA damage and stress were consistent with changes in laying rate. The abundance of Lactobacillus was related to the expression of *ANGPTRL1*, *ANGPTRL2*, *ANGPT1L*, and *NOXO1* in the duodenum; *Muricomes* was correlated significantly with *NFKBIZ*, *LYG2*, and *IRG1L* expression in the jejunum; and *Campylobacter* was correlated positively with the expression of *KMT2A* and *USF3* in the ileum. These results indicated that the intestinal microbiota and host gene expression may influence egg production jointly.

## 1. Introduction

The chicken (*Gallus gallus*) is an important agricultural and avian-model species, and it is a major source of protein worldwide [1]. The egg is rich in nutrients and vitamins, and the excellent performance of egg-laying could promote chicken genetic progress. It is therefore essential to promote the egg-laying rate for chicken breeders. The intestine, which is composed of the duodenum, jejunum, and ileum, is the central organ of digestion and absorption, where each segment performs unique functions. Intestinal dysfunction can lead to an uptake disorder of yolk precursors and a decreased egg-laying rate [2,3].

The composition of the microbiota in the digestive tract of chickens has been shown conclusively to be closely related to both host production and health [4,5]. Previous studies showed the gut flora community became stable during peak- and late-laying periods [6], while the different hormone levels could change the gut’s microbial community [7]. The abundance of *Lactobacillus*, *Lactococcus*, and *Bifidobacterium* was higher in chickens with higher laying rates than in hens with lower performance, and transplanting fecal flora from the high laying rate group could improve the laying rate [8]. Videnska et al. found that microbial diversity in the chicken gut increased after laying, and several bacteria that did not exist before laying colonized the gut after laying, such as Synergistetes, Fusobacteria, and Elusimicrobia, although their abundances were lower than the dominant flora [9]. During the peak-laying period, the level of Firmicutes decreased gradually, and the abundance of Bacteroidetes increased. In contrast, the abundance of Bacteroidetes exceeded that of Firmicutes and became the dominant phylum during the late-laying period [6]. Expect the influence of intestinal flora on chicken laying performance; egg quality was also affected. *Salmonella enteritidis* could enter the fallopian tube through the cloaca, change the expression of *TLR*, *NLR*, *AvBD,* and some cytokines in the fallopian tube, and affect the weight, shell strength, albumen height, and yolk color of the egg, leading to a decline in egg quality [10]. *Salmonella Enteritidis* infection decreased steroidogenesis and the development of granulosa cells in hens and further reduced egg production [11]. Recent studies have shown that dietary supplementation with *Clostridium butyricum* can accelerate fatty acid oxidation, reshape the gut microbiota and bile acid profile of laying hens, improve egg yolk color, and improve feed efficiency during the late laying period [12].

The gut microbiota influences host behavior by changing specific organs. The weight of the intestines in sterile chickens decreased, and the intestinal wall became thinner than controls [13,14]. Under sterile conditions or decreased abundance of gut microbes, hens showed shorter intestinal villi and shallower crypts, which may affect digestion negatively [14]. The increased abundance of *Clostridium perfringens* increased the crypt depth of the intestine, decreased the villus-to-crypt ratio, and finally resulted in a reduced laying rate [15]. Gut microbes can also interact with distal host organs, such as the hypothalamus, the liver, and the ovary. Gut microbes can degrade proteins and carbohydrates to produce serotonin, which promotes peristalsis of the small intestine and affects appetite and aggression behavior [16]. The portal venous circulation system is the basis of hepato–intestinal circulation, and disturbance of the gut microbiota can destroy the gut barrier so that harmful ingredients can be transferred to the liver and cause hepatic damage based on the hepatic portal system [17]. gmGUS (β-Glucuronidase) from the gut microbiota activated conjugated estrogen in the gut, and recycled estrogen was absorbed by the gut and participated in biological processes [18].

Previous studies have examined the gut microbiota during egg-laying, but their impact on gut function and the interaction between host and microbe are still unclear. In this research, we used microbiome and transcriptome analyses to investigate the changes in microbiota and hosts during egg-laying (Figure S1). We studied the factors that affect the variation in egg-laying systematically and provided a scientific basis for a better understanding of the regulation of egg production traits in chickens.

## 2. Materials and Methods

### 2.1. Animals

A total of 60 white-feathered breeder chickens (Guangdong Wens South Poultry breeding Co., Ltd., Yunfu, China) were selected randomly from 20,000 chickens, 20 individuals for each period. At pre- (20 w of age), peak- (30 w), and late- (58 w) laying periods, hens’ duodenums, jejunums, and ileums were collected for transcriptome sequencing. Intestinal contents were isolated from these three gut sites and were subjected to 16s rRNA sequencing and metagenomic sequencing. The determination and isolation of duodenums, jejunums, and ileums were referred to in a previous study [19].

### 2.2. Transcriptome Sequence and Data Analysis

The RNA extraction from the duodenum, jejunum, and ileum of chickens from different stages was performed as in our previous study [20]. Six individuals were sequenced for each stage. Using a Ribo-ZeroTM Magnetic Kit to separate mRNA from total RNA (Epicentre, Madison, WI, USA), library construction was based on a Hieff NGS^®^ Ultima Dual-mode RNA Library Prep Kit (Yeasen, Shanghai, China). Paired-end sequencing was performed using an Illumina Nova-Seq 6000 sequencing platform by Novogene Biotechnology Co. (Beijing, China).

Acquired raw reads were filtered through Fastp to remove low-quality reads and calculate the Q20, Q30, and GC content. Clean reads were aligned with the chicken reference genome (GRCg7b, GCF_016699485.2) using Hisat2 (2.2.1). We used Kallisto (0.44.0) to obtain transcripts per kilobase million (TPM) values to quantify the level of transcripts. Benjamini and Hochberg’s procedure was adopted to calculate the *p* value. Differentially expressed analysis was performed using the DESeq2 R package (v1.42.0), and genes with an adjusted *p*-value < 0.05 and |log2 fold change| >1 were considered differentially expressed genes (DEGs). Gene Ontology (GO) analysis was conducted using the online software DAVID (v2023q4) (https://david.ncifcrf.gov/summary.jsp (accessed on 22 February 2024)), and Kyoto Encyclopedia of Genes and Genomes (KEGG) pathway analysis was performed using the R package ClusterProfiler (v4.10.0). To cluster and to visualize the time series pattern of gene expression better, we used the ClusterGVis package in R software (v4.3.2). Psych (v2.4.3) was used to calculate the correlation between differentially expressed genes and the intestinal microbiota.

### 2.3. Microbial DNA Extraction and Microbiome Sequencing

Total genomic DNA of the gut contents (i.e., duodenum, jejunum, and ileum) of 60 chickens (i.e., 20 pre-laying hens, 20 peak-laying hens, and 20 late-laying hens) was extracted, and we used 1% agarose gel to detect the DNA quality. The PCR library construction for sequencing 16s rRNA followed previous work [21]. The V3-V4 region of the 16s rRNA gene was amplified using primers 341F (5′-CCTAYGGGRBGCASCAG-3′) and 806R (5′-GGACTACNNGGGTATCTAAT-3′). The library was sequenced on an Illumina NovaSeq platform, and 250-bp paired-end reads were generated. EasyAmplicon was used to perform detailed 16s rRNA sequencing analysis [22]. Vsearch (v2.14.1) and Usearch (v11.0.667) software were utilized for primer trimming, filtering, dereplication, and denoising to obtain clean amplicons, which were subsequently used for taxonomic annotation with the SILVA database (release 132_99). Alpha and beta diversities were assessed using Usearch. Alpha diversity was computed and displayed using Shannon and richness indices to characterize the abundance and diversity of microbial communities, while constrained principal coordinates analysis (cPcoA) was employed to examine beta diversity, indicating comparisons of intestinal microbiota among different egg-laying periods. Differential microbe detection between different groups was performed using STAMP (v2.1.3) software. Additionally, linear discriminant analysis (LDA) coupled with linear discriminant analysis effect size (LEfSe) (https://www.bic.ac.cn/BIC/#/analysis?page=b%27MzY%3D%27 (accessed on 22 February 2024)) was employed to identify differentially abundant intestinal microbiota across different egg-laying periods.

After quality control, a total of 0.2 μg of DNA was fragmented to an average size of approximately 400 bp using the Covaris M220 system (Covaris, Woburn, MA, USA) for paired-end library construction. The paired-end library was constructed using the NEXTFLEX™ Rapid DNA-Seq Kit (Bioo Scientific, Austin, TX, USA) following the manufacturer’s protocols. Adapters containing a full complement of sequencing primer hybridization sites were ligated to the blunt ends of the fragments. The paired-end library was sequenced on the Illumina NovaSeq platform (Illumina Inc., San Diego, CA, USA) at Majorbio Bio-Pharm Technology Co., Ltd. (Shanghai, China), using the NovaSeq 6000 S4 Reagent Kit v1.5 (300 cycles) according to the manufacturer’s instructions (www.illumina.com (accessed on 22 February 2024)). In total, 177 metagenomic datasets were successfully produced, comprising 60 duodenum content, 58 jejunum content, and 59 ileum content. However, the library construction failed for three samples (two jejunum samples and one ileum sample) due to low DNA quality. The metagenomic data obtained from chicken intestinal microbiota were analyzed using the free online platform of Majorbio Cloud Platform (www.majorbio.com (accessed on 22 February 2024)). Paired-end raw reads underwent adapter trimming and removal of low-quality reads (length < 50 bp or with a quality value < 20 or containing N bases) using fastp (v0.20.0). Reads were then aligned to the chicken genome using BWA (v0.7.9a), and any hits associated with the reads and their mate pairs were subsequently removed. Metagenomic data were assembled using MEGAHIT (v1.1.2), which employs succinct de Bruijn graphs for assembly [23]. Contigs with a length ≥ 300 bp were selected as the final assembling result, and then the contigs were adopted for gene prediction and annotation. Open reading frames were predicted for each assembled contig using Prodigal, and the ORFs > 100 bp were translated into amino acid sequences using the NCBI translation table (https://www.ncbi.nlm.nih.gov/Taxonomy/taxonomyhome.html/index.cgi?chapter=tgencodes#SG1 (accessed on 22 February 2024)) [24]. The construction of the non-redundant gene catalog was based on CD-HIT (v4.6.1) with 90% sequence identity and 90% coverage [25]. Representative sequences from the non-redundant gene catalog were aligned to the NR database with an e-value cutoff of 1 × 10^−5^ using Diamond (v0.8.35) to acquire taxonomic annotations. Diamond was also utilized for Cluster of Orthologous Groups of Proteins (COG) annotation of the representative sequences based on the eggNOG database. Additionally, KEGG annotation was conducted using Diamond against the Kyoto Encyclopedia of Genes and Genomes database (http://www.genome.jp/kegg/ (accessed on 22 February 2024)) with an e-value cutoff of 1 × 10^−5^.

### 2.4. Ethical Approval

The Nanjing Agricultural University Animal Care and Use Committee approved all procedures.

### 2.5. Data Availability

The sequencing data used in this study are available at the China National Center for Bioinformation (https://www.cncb.ac.cn/ (accessed on 22 February 2024)) and under Genome Sequence Archive (GSA) CRA016349 (16s rRNA sequencing data), CRA016365 (Metagenomic sequencing data), and CRA016325 (RNA-seq data).

## 3. Results

### 3.1. 16s rRNA Sequencing Information of the Duodenum, Jejunum, and Ileum Content during Egg-Laying Periods

To explore the change in intestinal flora during egg-laying periods, we collected the microbial samples of the duodenum, the jejunum, and the ileum from pre- (20 weeks), peak- (30 weeks), and late-laying (58 weeks) periods (*n* = 20). The 16s rRNA sequencing of 180 samples generated a total of ~25.72 M high-quality reads, which averaged 142.66 K reads per sample (Table S1).

### 3.2. Dynamically Microbial Changes in Duodenum during Egg-Laying Periods

**Figure 1:**
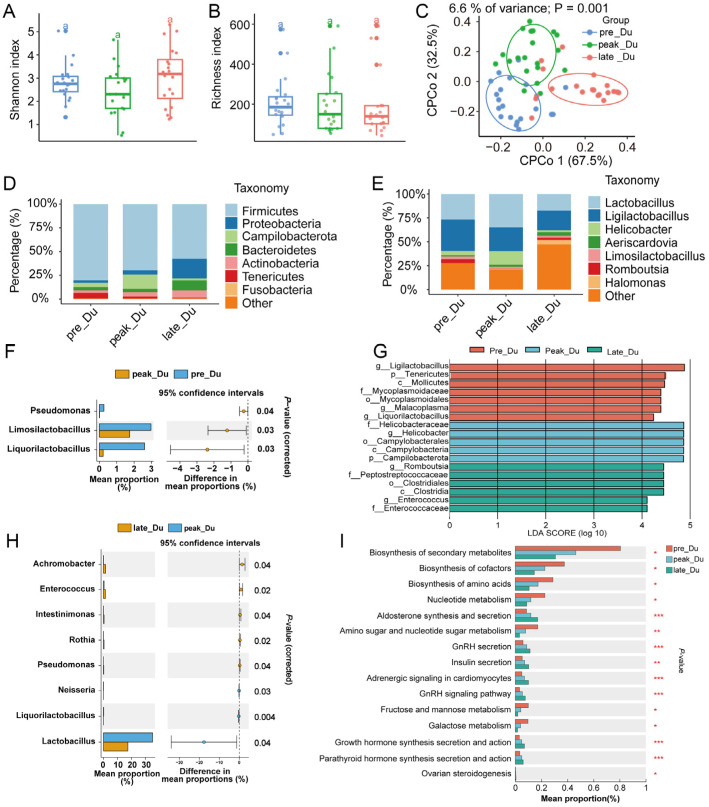
The (**A**) Shannon and (**B**) richness indexes of the duodenal microbes from different egg-laying periods in hens. (**C**) Constrained principal coordinates analysis (cPCoA) based on weighted Bray-Curtis distance in duodenum. The microbial composition of duodenum at the (**D**) phylum and (**E**) genus levels. (**F**) Differential duodenal microbes between pre- and peak-laying periods and (**H**) between peak- and late-laying periods. (**G**) Microbial biomarkers from duodenum during laying (LDA > 4, *p* < 0.05). (**I**) KEGG pathway identification of duodenum during laying periods. ^a^ Columns with same superscripts meant no significance (*p* > 0.05). * *p* < 0.05, ** *p* < 0.01, *** *p* < 0.001.

The Richness index showed that the abundance of duodenal microbiota decreased in the late-laying period, and the Shannon index suggested that the duodenal microbial diversity died during egg-laying (*p* > 0.05) (Figure 1A,B). Constrained principal coordinates analysis (cPCoA) showed a significant separation among duodenal microbiota from different egg-laying periods (Figure 1C). The most abundant phyla (top eight from all samples) and genera (top eight from all samples) were exhibited with stack bar charts (Figure 1D,E). Firmicutes (69.2%), Proteobacteria (9.26%), Campilobacterota (7.26%), and Bacteroidetes (5.76%) were the four most dominant phyla in the duodenum, and they dominated the microbial community by more than 91% (Figure 1D). *Lactobacillus* and *Ligilactobacillus* were the most common genera during egg-laying periods (Figure 1E).

Differential analysis using STAMP (v2.1.3) software suggested that the relative abundance of three genera changed significantly between pre- and peak-laying periods; seven differential microbes were identified in peak- and late-laying periods (Figure 1F,H). Based on the linear discriminant analysis (LDA) effect size (LEfSe) method, 11 different microbes were found during the egg-laying period (Figure 1G).

We used metagenomic sequencing to understand the microbial functional changes during egg-laying. KEGG analysis revealed that the relative abundances of biosynthesis of secondary metabolites, cofactors, amino acids, fructose, mannose metabolism, and galactose metabolism decreased with an increase in laying days. In contrast, aldosterone synthesis and secretion levels, GnRH secretion, insulin secretion, growth hormone synthesis secretion and action, and ovarian steroidogenesis increased after laying (Figure 1I). These results indicated that microbes in the duodenum were altered dynamically; the microbial functional changes may have resulted in the laying rate of chickens.

### 3.3. Transcriptome Analysis of Duodenum during Egg-Laying Periods

**Figure 2:**
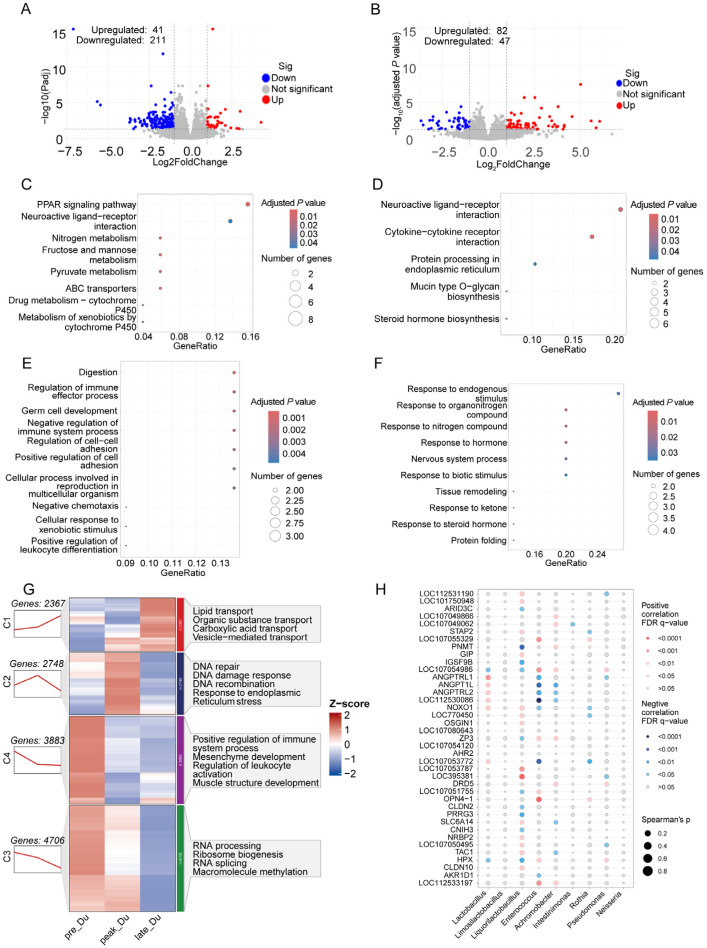
Volcano plots that display differentially expressed genes (DEGs) in duodenum between peak- and pre-laying periods in hens (**A**), KEGG (**C**), and Go (**E**) analysis, which suggest the function of DEGs. Identification of DEGs between late- and peak-laying periods (**B**), KEGG (**D**), and GO (**F**) analysis detected the functional changes in DEGs. (**G**) Sequential analysis of duodenal transcriptomics among three egg-laying periods. (**H**) Conjoint analysis of differential duodenal microbiota and genes.

Through differential analysis, a total of 41 significantly up-regulated and 211 down-regulated genes were identified in the duodenum from the peak-laying period to the pre-laying period (Figure 2A). KEGG analysis indicated that the DEGs involved in PPAR signaling pathways, neuroactive ligand–receptor interaction, nitrogen metabolism, and GO analysis influenced digestion, regulation of immune effector processes, germ cell development, and cellular response to xenobiotic stimuli (Figure 2C,E). A total of 129 DEGs were detected between the late- and peak-laying periods, which involved neuroactive ligand–receptor interaction, protein processing in the endoplasmic reticulum, response to endogenous stimulus, and response to hormones (Figure 2B,D,E).

Sequential analysis suggested that genes from the duodenum (TPM > 0.5) had different expression modes, which were divided into four clusters. The expression of genes from cluster 1 showed a continuously increasing trend; these genes were enriched in lipid transport (*APOA1*, *APOB*, *FZD4*), organic substance transport (*ACTN4*, *ANXA5*, *APOA1*, *APOB*), carboxylic acid transport (*LBFABP*, *NOS2*, *PQLC2*), and vesicle-mediated transport (*APOA1*, *B2M*, *CCDC93*). A total of 2748 genes were in cluster 2, and their expression was up-regulated during the peak-laying period and down-regulated in the late-laying period; they were involved in response to DNA damage and to endoplasmic reticulum stress. Genes from clusters 3 and 4 showed decreased expression during laying, which regulated mesenchyme development, positive regulation of the immune system process, and ribosome biogenesis (Figure 2G).

To gain insight into the connection between microbes and host gene expression, we utilized duodenal microbiota from 16s rRNA sequencing and the top 40 DEGs during laying to conduct a correlation analysis. Our findings revealed a significant positive correlation between the expression of *ANGPTRL1*, *ANGPTRL2*, *ANGPT1L*, and *NOXO1* and the relative abundance of *Lactobacillus*. Additionally, the levels of *Enterococcus* and *Achromobacter* were correlated with the expression of *ZP3* (Figure 2H).

### 3.4. Dynamically Microbial Changes in Jejunum during Egg-Laying Periods

**Figure 3:**
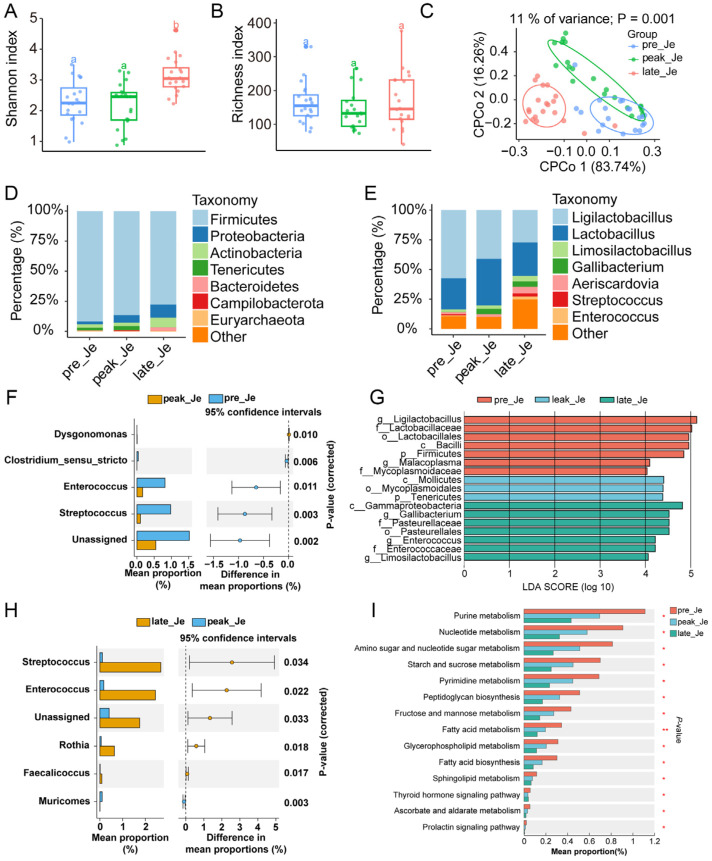
The Shannon (**A**) and richness (**B**) indexes of the jejunal microbes from different egg-laying periods of hens. Jejunal cPCoA analysis of jejunum from different egg-laying periods (**C**). The microbial composition of jejunum at the phylum (**D**) and genus level (**E**). Identification of differential microbes in pre- and peak-laying periods (**F**) and between peak- and late-laying periods (**H**). (**G**) Biomarkers of jejunum during different egg-laying periods (LDA > 4, *p* < 0.05). (**I**) KEGG pathway identification of jejunal microbiota during egg-laying. ^a,b^ Columns with different superscripts differ significantly (*p* < 0.05). * *p* < 0.05, ** *p* < 0.01.

The Shannon index suggested microbial diversity increased significantly during the late-laying period, while the richness index of jejunal microbes decreased during the peak-laying period (Figure 3A,B). cPCoA analysis indicated that the microbial community changed significantly (*p* < 0.05), but the microbial community in the jejunum from the pre-laying period was similar to the peak-laying period (Figure 3C). Firmicutes (85.39%), Proteobacteria (6.48%), Actinobacteria (4.60%), and Tenericutes (1.85%) were the four most abundant phyla, and *Ligilactobacillus* (41.91%), *Lactobacillus* (31.11%), *Limosilactobacillus* (3.23%), and *Gallibacterium* (3.11%) were the four most abundant genera in the jejunum during egg-laying (Figure 3D,E).

LEfSe (LDA > 4, *p* < 0.05) analysis showed there were 17 significantly different microbes in pre-, peak-, and late-laying periods (Figure 3G). Differential analysis revealed that the relative abundance of four microbes changed significantly between pre- and peak-laying, and the relative abundance of five differential microbes changed significantly during late- and peak-laying (Figure 3F,H). KEGG analysis from jejunal metagenomic sequencing showed that the relative abundance of purine, nucleotide, starch, sucrose, and fatty acid metabolism decreased with the increase in egg-laying days (Figure 3I).

### 3.5. Transcriptome Analysis of Jejunum during Egg-Laying Periods

**Figure 4:**
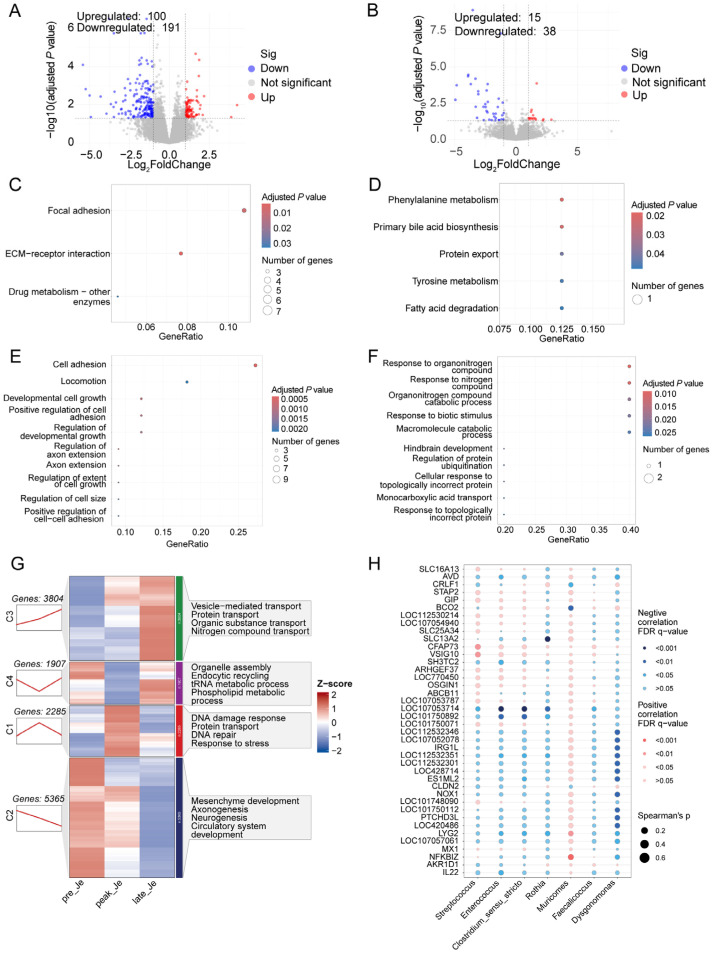
Volcano plots that display differentially expressed genes (DEGs) in jejunum between peak- and pre-laying periods of hens (**A**), KEGG (**C**), and Go (**E**) analysis, which suggest the function of DEGs. Identification of DEGs between late- and peak-laying periods (**B**), KEGG (**D**), and GO (**F**) analysis detected functional changes in DEGs. (**G**) Sequential analysis of duodenal transcriptomics among three egg-laying periods. (**H**) Conjoint analysis of differential jejunal microbiota and genes.

A total of 291 DEGs were identified in the jejunum during pre- and peak-laying periods; KEGG and GO analysis showed that these genes were involved in focal adhesion, cell adhesion, and ECM-receptor interactions (Figure 4A,C,E). Compared with the peak-laying period, 15 up-regulated and 38 down-regulated genes were detected in the jejunum from the late-laying period (Figure 4B). These genes regulate phenylalanine metabolism, primary bile acid metabolism, tyrosine metabolism, response to organonitrogen compounds, and response to biotic stimulus (Figure 4D,F).

Sequential analysis suggested that the genes in the jejunum had four different expression modes. A total of 2285 genes were placed into cluster 1, which involved responses to DNA damage, stress, and DNA repair. The expression of genes from cluster 2 decreased continuously during egg-laying; these genes regulated mesenchyme development, axonogenesis, and neurogenesis. A total of 3804 genes whose expression increased continuously during laying were placed into cluster 3, which is involved in vesicle-mediated transport (*TMEM175*, *TMEM251*, and *TRAPPC11*), transport of organic substances (*TST*, *ANXA5*, *STAM2*, and *SCP2*), and nitrogen compounds (*TST*, *STAM2*, and *LIN7C*) (Figure 4G). Conjoint analysis revealed that the relative abundance of *Muricomes* correlated positively with the levels of *NFKBIZ*, *LYG2*, *IRG1L*, and *NOX1*; *Streptococcus* was correlated with *CFAP73*, *VSIG10*, and *GIP* (Figure 4H).

### 3.6. Dynamically Microbial Changes in Ileum during Egg-Laying Periods

**Figure 5:**
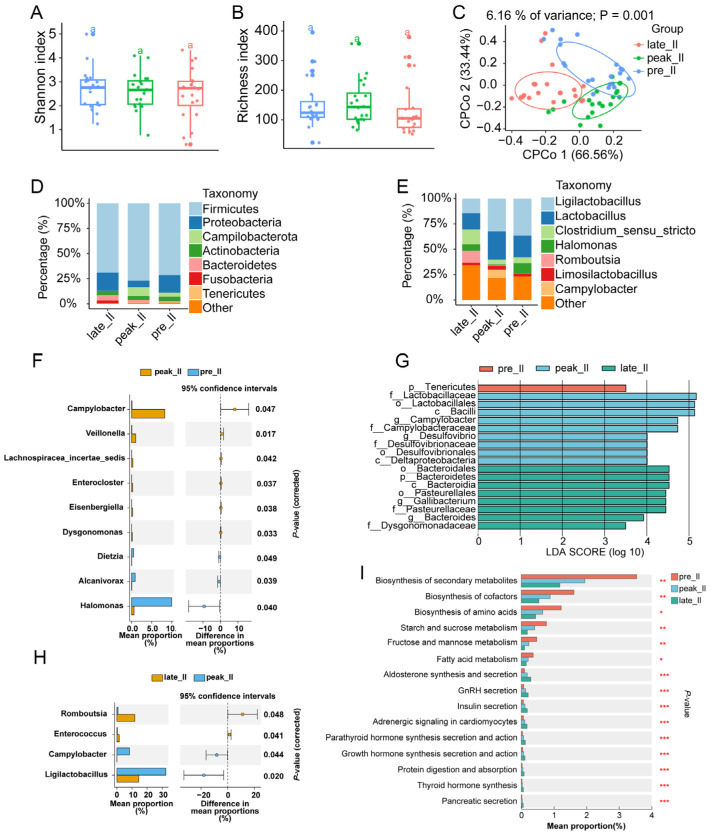
Shannon (**A**) and richness (**B**) indexes in the ileal microbiota during egg-laying periods in hens. cPCoA analysis based on weighted Bray-Curtis distance in the ileum (**C**). The microbial composition of duodenum at the phylum (**D**) and genus level (**E**). Differential ileal microbes between pre- and peak-laying periods (**F**) and differential flora in peak- and late-laying periods (**H**). (**G**) Microbial biomarkers from duodenum during laying (LDA > 4, *p* < 0.05). (**I**) KEGG pathway identification of duodenum during laying periods. ^a^ Columns with same superscripts meant no significance (*p* > 0.05). * *p* < 0.05, ** *p* < 0.01, *** *p* < 0.001.

Alpha diversity indicated that the microbial diversity in the ileum remained stable during egg-laying, and the richness of the ileal microbiota increased during the peak-laying period and decreased during the late-laying period (*p* > 0.05). Based on cPCoA analysis, the microbial community changed significantly during egg-laying, and the community of ileal microbiota from the peak-laying period was similar to the pre-laying period (Figure 5C). Firmicutes (72.51%), Proteobacteria (14.06%), Campilobacterota (4.28%), and Actinobacteria (4.09%) were the most abundant phyla, and *Ligilactobacillus* (27.80%), *Lactobacillus* (21.72%), *Clostridium_sensu_stricto* (8.47%), *Halomonas* (5.67%), and *Romboutsia* (4.27%) were the most abundant genera in the ileum during egg-laying periods (Figure 5D,E).

Nine differential microbes were identified in the ileum during pre- and peak-laying periods (Figure 5F), and the relative abundance of four microbes changed significantly during the peak- and late-laying periods (Figure 5H). Using LEfSe analysis, 18 microbial biomarkers were detected during egg-laying (Figure 5G). KEGG analysis from metagenomic sequencing suggested that the relative abundances of biosynthesis of secondary metabolites, cofactors, amino acids, and fatty acid metabolism decreased with increased laying days. In contrast, the levels of aldosterone synthesis and secretion, GnRH secretion, insulin secretion, parathyroid hormone synthesis secretion and action, and the synthesis, secretion, and action of growth hormones increased during egg-laying periods (Figure 5I).

### 3.7. Transcriptome Analysis of Ileum during Egg-Laying Periods

**Figure 6:**
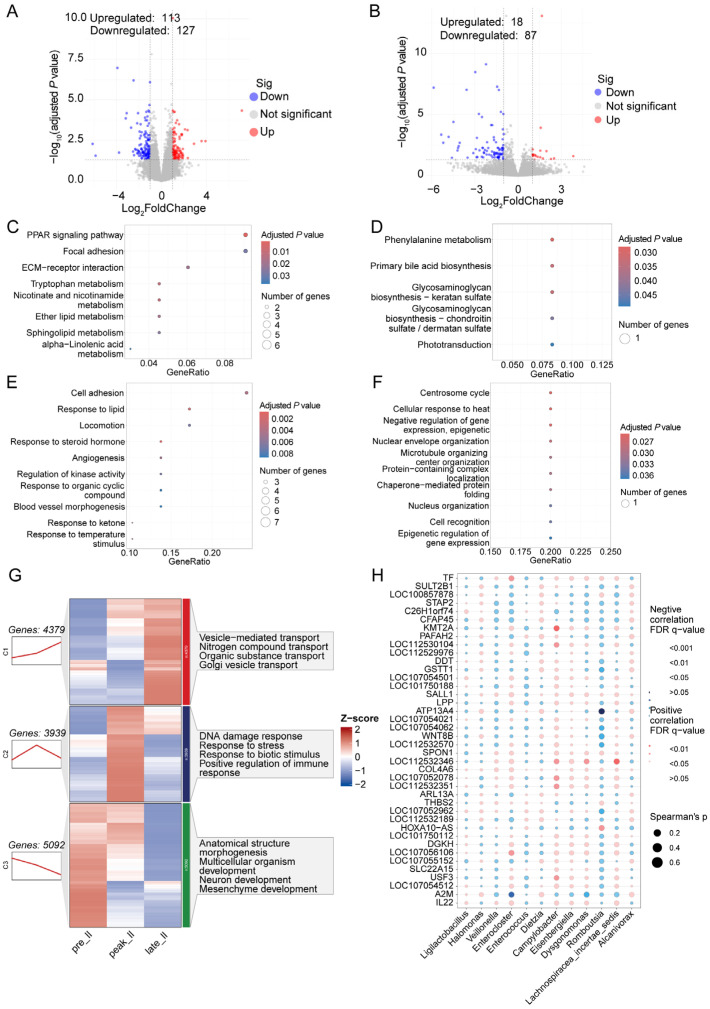
Volcano plots that display differentially expressed genes (DEGs) in the ileum between peak- and pre-laying periods of hens (**A**), KEGG (**C**), and GO (**E**) analysis, which suggest the function of DEGs. Identification of DEGs between late- and peak-laying periods (**B**), KEGG (**D**), and GO (**F**) analysis detected the functional changes in DEGs. (**G**) Sequential analysis of ileal transcriptomics among three egg-laying periods. (**H**) Conjoint analysis of differential ileal microbiota and genes.

Differential analysis of the ileum during pre- and peak-laying periods detected 240 DEGs that were involved in the PPAR signaling pathway, focal adhesion, ECM-receptor interaction, response to lipids, and response to steroid hormones (Figure 6A,C,E). A total of 105 DEGs in the ileum were identified during peak- and late-laying periods (Figure 6B); these genes regulated phenylalanine metabolism, biosynthesis of primary bile acids, cellular response to heat, and negative regulation of gene expression (Figure 6D,F).

Genes in the ileum from different egg-laying periods were divided into three clusters according to different expression modes. Cluster 1 consisted of 4379 genes that were up-regulated continuously during egg-laying; these genes regulated vesicle-mediated transport (*CCDC93*, *COPE*, and *EXOC8*), nitrogen compound transport (*AACS*, *ACTN4*, and *AKTIP*), and Golgi vesicle transport (*CCDC93*, *COPE*, and *EXOC8*). A total of 3939 genes were allocated to cluster 2, and their expression increased during the peak-laying period and decreased during the late-laying period; these genes involved response to DNA damage, response to stress, and positive regulation of immune response. Cluster 3 included 5092 genes that decreased continuously during egg-laying and influenced the morphogenesis of anatomical structure, neuron development, and mesenchyme development.

Through conjoint analysis between differential microbiota and genes in the ileum, the levels of *KMT2A* and *USF3* were positively correlated with the abundance of *Campylobacter*. *Enterocloster* was correlated with the *TF* gene, and *GSTT1*, *WNT8B*, and *ATP13A4* were correlated negatively with *Romboutsia* (Figure 6H).

## 4. Discussion

The intestine consists of the duodenum, jejunum, and ileum, which are the primary sites of digestion, although the three segments have different functions. During egg-laying periods, fluctuating hormones influence the microbial community in the chicken gut [7]. gmGUS from flora can uncouple the coupling estrogen in the intestine, and the activated estrogen can affect the host estrogen level by entering the circulation system [18]. The gut microbiota and host hormone levels interacted and mutually contributed to host behavior. Previous studies mainly explored the dynamic change in intestinal microbiota during egg-laying, but the interaction with host expression was unclear. Therefore, we collected intestinal tissues and microbes from pre-, peak-, and late-laying periods to acquire a more comprehensive understanding of egg-laying based on microbiome and transcriptome analyses.

### 4.1. Changes in Duodenal Microbiota and Gene Expression during Egg Laying

The richness of duodenal flora decreased during egg-laying while the diversity increased. Firmicutes, Proteobacteria, Campilobacterota, and Bacteroidetes were the dominant phyla. The abundance of Firmicutes decreased with increased egg-laying days, the levels of Proteobacteria and Bacteroidetes increased during the late-laying period, and Campilobacterota also increased during the peak-laying period; these results were similar to a previous study [7]. Bacteroidetes and Firmicutes are able to increase the body weight of the host by affecting the ability to harvest energy from the diet [21], and body weight has a negative association with egg production [26].

Differential analysis showed that the relative abundances of *Pseudomonas*, *Limosilactobacillus*, and *Liquorilactobacillus* were higher during pre-laying than during peak-laying. *Pseudomonas*, which was a dominant bacteria during embryonic development, digested cellulose through the vitamin breakdown pathway [27]. The relative abundance of *Limosilactobacillus* increased significantly in the cecum of chickens with high feed efficiency and was correlated with nutrient absorption [28]. *Liquorilactobacillus* is a novel genus with probiotic functions that is capable of regulating the intestinal microbiota, relieving enteritis, and exhibiting high antimicrobial activity [29]. The increased abundance of these microbes may promote egg-laying. The abundances of *Achromobacter* and *Enterococcus* increased, and the level of *Lactobacillus* decreased during late-laying periods. *Lactobacillus* is a probiotic and feed additive that improves laying performance and changes the composition of microbes in the feces [30]. *Enterococcus* and *Achromobacter* are opportunistic pathogens that cause poultry diseases [31]. The decreased concentration of beneficial bacteria and the increased abundance of harmful bacteria may reduce the egg-laying rate during the late-laying period. KEGG analysis from metagenomic sequencing showed the relative frequency of biosynthesis of secondary metabolites, cofactors, and amino acids decreased. The levels of synthesis and secretion of aldosterone, GnRH secretion, insulin secretion, synthesis, secretion, and action of growth hormones, and ovarian steroidogenesis increased. Though hormone levels may increase during the late-laying period, the reduced substances cannot satisfy nutritional requirements, resulting in a decreased laying rate during the late-laying period.

DEGs during the pre- and peak-laying periods were involved in PPAR signaling pathways and regulation of immune effector processes. The PPAR signaling pathway has several functions, which include regulation of lipid metabolism and immune regulation [32]. The increased laying rate depends on the function of the PPAR signaling pathway. DEGs during peak- and late-laying periods regulate neuroactive ligand—receptor interaction and response to hormones, which indicated that the nervous and endocrine systems changed during the late-laying period. Sequential analysis revealed that continuously up-regulated genes in the duodenum during the egg-laying period involved several transport processes, and down-regulated genes regulated the immune system, mesenchyme development, and muscle structure development. These genes exhibited a similar expressed trend during egg-laying related to DNA repair and response to DNA damage. The transport process of the intestine is necessary for egg production; the reduced development of intestinal structure may be due to aging, and the change in response to DNA damage was perhaps attributed to the stress of the duodenum from the changed laying rate. These changes in the duodenum may lead, in combination, to a changed egg-laying rate.

Through conjoint analysis between differential microbes and duodenal genes, we found that Lactobacillus was related to the expression of *ANGPTRL1*, *ANGPTRL2*, *ANGPT1L*, and *NOXO1*, and the level of *ZP3* was correlated with *Enterococcus* and *Achromobacter*. *ANGPTRL1*, *ANGPTRL2*, and *ANGPT1L* are angiopoietin-related proteins that can affect angiogenesis. Knockout of *NOXO1* increases the proliferation of intestinal epithelial cells, affects epithelial homeostasis, and maintains homeostasis [33]. Supplementation with dietary Lactobacillus improved egg-laying performance and eggshell quality and reduced the feed conversion ratio during the late-laying period [34]. Hong et al. found that Lactobacillus feeding regulated the activity of intestinal stem cells and improved egg weight, egg mass, and egg-laying rate [35]. Therefore, we speculated that *Lactobacillus* affected duodenal angiogenesis and proliferation of epithelial cells to change duodenal functions, which eventually altered the egg-laying rate. *Enterococcus* and *Achromobacter* were opportunistic pathogens, though rare functions of zona pellucida glycoprotein 3 (*ZP3*) in the intestine have been reported, and its homologous gene, *ZP4*, could be anti-inflammatory in the intestine [36]. These results suggested duodenum and microbiota interacted, microbiota influenced gut development, and duodenum could affect microbiota during egg-laying periods.

### 4.2. Changed Microbiota and Gene Expression in Jejunum during Egg Laying

The microbial composition of the jejunum was similar to that in the duodenum at the phylum level. The abundant changes in Firmicutes and Proteobacteria were identical with the duodenum during egg-laying, and Actinobacteria and Tenericutes became the third and fourth most dominant bacteria, which may be due to the different functions between the duodenum and the jejunum. Differential analysis found that the level of *Dysgonomonas* increased significantly during the peak-laying period, and the levels of *Rothia* and *Enterococcus*, which increased during the late-laying period, are opportunistic pathogens that can induce various infections that may result in a decreased laying rate during the late-laying period [37]. *Dysgonomonas* contains multiple genes for lignocellulolytic enzymes and contains complete genes for lignocellulose degradation, acetate and lactate pathways, and pathogen defense; these genes may promote an increased egg-laying rate due to lignocellulose degradation and immune function [38]. KEGG analysis based on metagenomics sequencing suggested that the relative abundance of purine metabolism, nucleotide metabolism, starch, sucrose metabolism, and fatty acid metabolism decreased during egg-laying, and reduced uptake of nutrients from the jejunum eventually resulted in a decreased laying rate during the late-laying period.

DEGs in the jejunum during pre- and peak-laying periods were involved in focal adhesion, ECM-receptor interaction, growth of developmental cells, and axon extension; these functional changes may have influenced the development of the enteric nervous system. DEGs in the jejunum from peak- and late-laying were involved in phenylalanine metabolism, biosynthesis of primary bile acids, and response to biotic stimulus. Phenylalanine can combine bile acid and tyrosine to activate the farnesoid X receptor (FXR), which is increased in Crohn’s disease [39]. These results indicated that jejunal functions may be damaged and result in a decreased laying rate during the late-laying period. Sequential analysis of jejunal genes during egg-laying found similar results to those of the duodenum. Continuously up-regulated genes involved vesicle-mediated transport, protein transport, and organic substance transport; continuously down-regulated genes regulated mesenchyme development, axonogenesis, and circulatory system development. Genes that increased during the peak-laying period and decreased during the late-laying period were involved in DNA damage response, DNA repair, and stress response.

Conjoint analysis of differential genes and microbes found that the abundance of *Muricomes* was significantly correlated with the expression of *NFKBIZ*, *LYG2*, and *IRG1L*. The level of *Muricomes* was abundant in mice with colonitis [40], and it was associated with liver LDL-cholesterol, which increased in mice fed high-fat food [41]. The product encoded by *NFKBIZ* is one of the nuclear I kappa B proteins and an activator of IL-6 production. Mutations of *NFKBIZ* were common in animals with colitis [42]. Lysozyme g2 is encoded by the *LYG2* gene, which is a lysozyme capable of host defense against infection [43], and *IRG1L* is a marker gene of the innate immune system [44]. The increased abundance of *Muricomes* suggests an unhealthy state of the jejunum or disordered jejunal microbial community, which may result from a higher laying rate during the peak-laying period, and the jejunum relieves the adverse state through changing the expression of several genes related to immune function.

Combining the above results, we hypothesize that an increased laying rate may disturb the jejunal microbial community and increase the level of pathogens. Jejunum used several antimicrobial substances to relieve the threat from pathogens.

### 4.3. Changes in Ileal Microbiota and Gene Expression during Egg Laying

The results of 16s rRNA sequencing revealed that the richness and diversity of the ileal microbiota decreased during the late-laying period. The dominant phyla in the ileum were identical to those in the duodenum and jejunum. There were differences in the abundance of specific phyla, possibly due to diverse intestinal segment functions. Differential microbial analysis suggested that the abundances of *Campylobacter*, *Veillonella*, *Lachnospiracea_incertae_sedis, Enterocloster*, *Eisenbergiella*, and *Dysgonomonas* were greater in the ileum during the peak-laying period than during the pre-laying period.

*Veillonella*, which was first isolated from the cecal contents of chickens, reduced the colonization of *Salmonella* and was associated with the immunity of the chicken ileum barrier [45,46]. *Lachnospiracea_incertae_sedis* is a beneficial bacterium associated with piglets’ intestinal immunity after weaning [47]. The decrease in the relative abundance of *Lachnospiracea_incertae_sedis* in the gut was associated with feather-pecking behavior and immune stress, which may affect the central nervous system and immune system of chickens [48]. An increased abundance of these ileal microbiota may enhance immune function and protect the laying rate. Compared with the peak-laying period, the concentrations of *Romboustia* and *Enterococcus* increased, while *Campylobacter* and *Ligilactobacillus* decreased. *Romboutsia* was positively correlated with propionic acid, butyric acid, and cholic acid, which can regulate intestinal immunity and reduce intestinal inflammation in chickens [49]. The increased *Romboutsia* may balance the negative effect of *Enterococcus*, which is an opportunistic pathogen. The abundance of *Campylobacter*, which is a widespread pathogen in chickens that causes an immune response and leads to decreased egg quality, increased during peak-laying [50]. The growth of *Campylobacter* rectus was stimulated significantly with the supplementation of estradiol in the culture medium and host [51]. Changes in *Campylobacter* may result from the increased estradiol level during the peak-laying period [1].

KEGG analysis suggested that the relative abundance of biosynthesis of secondary metabolites, cofactors, and amino acids decreased. The levels of aldosterone synthesis and secretion, GnRH secretion, insulin secretion, the synthesis of parathyroid hormone, and growth hormone were enhanced during egg-laying. From the above results, we believe that changes in amino acids and hormones in the ileum microbiota influence egg-laying.

Through enrichment analysis, during the pre- and peak-laying periods, DEGs were involved in the PPAR signaling pathway, ether lipid metabolism, sphingolipid metabolism, and response to steroid hormone, which suggested that lipid metabolism and hormones changed during the peak-laying period. DEGs from the peak- and late-laying periods regulated phenylalanine metabolism and primary bile biosynthesis, which was similar to the results from the jejunum; this suggested consistent changes in function. Sequential analysis found similar results with duodenum and jejunum: up-regulated genes regulated several transport processes, down-regulated genes involved in the development of intestinal structure, and genes whose expression changed were consistent with the trend in egg-laying rate that influenced response to DNA damage. These results suggested the same functional changes within different intestinal segments. Conjoint analysis revealed that the relative abundance of *Campylobacter* was positively correlated with the expression of *KMT2A* and *USF3*. *Campylobacter* is a widespread pathogen in chickens, and severely influences human health [52], and previous studies suggested that the abundance of Campylobacter is influenced by the level of estradiol [1]. The *KMT2A* gene encodes a transcriptional coactivator that regulates gene expression during development, which influences interferon and function in inflammation [53]. *USF3* is an upstream transcription factor whose deficiency enhances glutamine-dependent survival and sensitivity to endoplasmic reticulum stress [54] and is involved in chicken immune function [55]. Therefore, we considered that a changed estradiol level increased pathogen abundance during the peak-laying period, and the ileum relieved the influence of pathogens through gut immunity.

As mentioned above, microbial function changed in the same intestinal segment during the different egg-laying periods; we also analyzed the intestine’s functional changes with longitudinal dimensions (Figure S2). The relative abundance of pathways that biosynthesize secondary metabolites, cofactors, and amino acids was higher in the jejunum and ileum than in the duodenum, which was gradually decreased with the increased egg-laying days that may be due to the aging of the intestine. The level of pathways for GnRH secretion, GnRH signaling pathway, growth hormone synthesis secretion, and action increased in peak- and late-laying periods. In addition, we also detected microbiota in the cecum during chicken egg-laying and the relative abundance of pathways of biosynthesis of secondary metabolites and amino acids was similarly decreased with increased laying days; the GnRH signaling pathway, steroid hormone biosynthesis, and the estrogen signaling pathway were increased significantly during the peak-laying periods. Functional changes in the duodenum, jejunum, ileum, and cecum suggested that microbiota residing in the gut may perform identical functions in chicken egg-laying.

## 5. Conclusions

The composition of the intestinal microbial community underwent alterations, which led to an increase in the abundance of pathways for synthesizing microbial hormones and a decrease in amino acid synthesis. During egg-laying, modifications in the expression of intestinal genes were observed that affected transport function, structural development, and the response to DNA damage. By analyzing the correlation between microbes and host gene expression, microbes in the host may have a collective impact on chicken egg-laying. These results provided more comprehensive knowledge of dynamically changed gut microbes and host gene expression during chicken egg-laying and suggested a novel method to promote egg production in the poultry industry.

## Data Availability

Sequencing data analyzed in this study are available by reasonable demand from the corresponding author.

## Supplementary Materials

The following supporting information can be downloaded at: https://www.mdpi.com/article/10.3390/ani14111529/s1, Figure S1: Overview of the integrated multi-omics workflow combining microbiome and transcriptomics.; Figure S2: The identified pathways among different intestinal segments during egg-laying periods; Table S1: Summary of 16S rDNA sequencing.
