# Supplementary material for: Dynamic Changes in Intestinal Gene Expression and Microbiota across Chicken Egg-Laying Stages
Source: Animals (Basel). 2024 May 22;14(11):1529. doi: 10.3390/ani14111529 (PMC11171086; doi:10.3390/ani14111529)

Figure. S1. Overview of the integrated multi-omics workflow combining microbiome and transcriptomics.

A

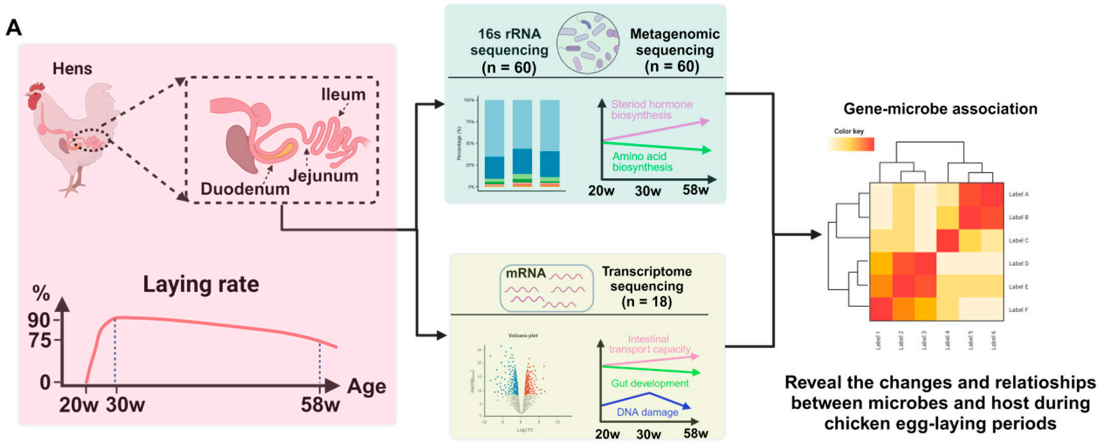

B

Sequencing Methods	Transcriptome sequencing			Microbiome sequencing (16s rRNA/Metagenomic sequencing)		
Sequencing samples	Intestine tissue			Intestinal content		
Intestinal segments	Duodenum	Jejunum	Ileum	Duodenum	Jejunum	Ileum
Pre-laying period	n = 6	n = 6	n = 6	n = 20	n = 20	n = 20
Peak-laying period	n = 6	n = 6	n = 6	n = 20	n = 20	n = 20
Late-laying period	n = 6	n = 6	n = 6	n = 20	n = 20	n = 20

A. The skeleton chart of the experiment; B. The samples information of sequencing in different groups.

Figure. S2. The identified pathways among different intestinal segments during egg-laying periods

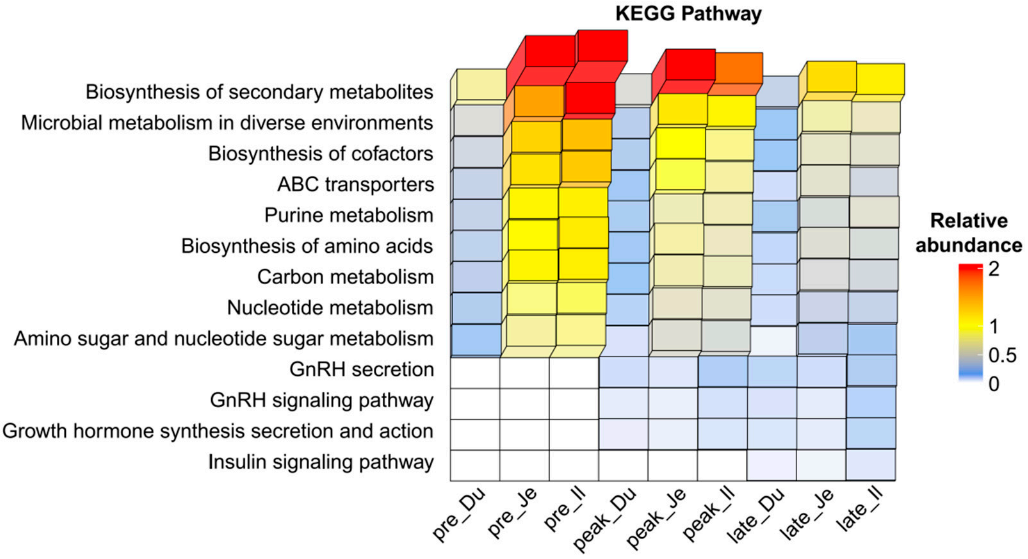

Supplement: Supplementary file 1 [file animals-14-01529-s001.zip › supplemental figures.pdf]
